# Extraction socket grafting using recombinant human bone morphogenetic protein-2-clinical implications and histological observations

**DOI:** 10.1186/s13104-021-05476-0

**Published:** 2021-02-15

**Authors:** Khushboo Jeevan Durge, Vidya Sudhindra Baliga, Shilpa Bangalore Sridhar, Prasad Vijayrao Dhadse, Gayatri Chandrakant Ragit

**Affiliations:** Department of Periodontics, Sharad Pawar Dental College Sawangi (Meghe), Datta Meghe Institute of Medical Sciences (Deemed To Be University), Wardha, Maharashtra India

## Abstract

**Objectives:**

Rehabilitation of edentulous ridges to promote the insertion of dental implants has been the key indicator for retaining osseous structures since tooth extraction. Recombinant Bone Morphogenetic Protein-2(rhBMP-2) is exploited for bone augmentation due to its osteoinductive capacity. The objective of the study to determine the effectiveness of bone induction for implant placement by rhBMP-2 delivered on beta-tricalcium phosphate graft (β-TCP) and PRF following tooth extraction.

**Results:**

Minimal changes in the width of the crestal bone relative to baseline values were found three months after socket grafting. A bone loss in the mesiodistal and buccolingual aspects of 0.6 ± 0.13 mm and 0.5 ± 0.13 mm was found, respectively. While drilling before the implant placement, the bone's clinical hardness evaluated through tactile was analogous to drilling into spruce or white pine wood. Total radiographic bone filling was seen in 3 months and no additional augmentation was needed during implant placement. Besides, histology shows no residual graft of bone particles. Therefore, the data from this study demonstrated that the novel combination of rhBMP-2 + β-TCP mixed with PRF has an effect on de novo bone formation and can be recommended for socket grafting before implant placement.

## Introduction

Endosseous implants have currently been the therapy option for the treatment of an edentulous site [1–3]. However, after extraction, owing to osteoclastic activity, the alveolar process undergoes resorption, leading to reduced alveolar ridge measurements in both the vertical and horizontal planes [4]. Alveolar ridge alterations ranging from 40–60% tend to occur as early as three months after extraction [5]. The altered height and width have practical and aesthetic issues that result in impaired recovery. Within 12 months after removal of the tooth, non-grafted edentulous sites can lose up to fifty percent of ridge width [6]. An average reduction reported in the width and height of grafted edentulous site`s is minimal compared to non-grafted site`s, with most cases requiring no additional augmentation to have the implants placed [7]. Therefore, surgical approaches aiming to maintain ridge volume by preservation before implant placement are deemed useful. Platelet-rich fibrin (PRF) comprises of fibrin matrix enriched with platelets and growth factors. Hence it is a powerful bioscaffold for tissue regeneration [8]. Alloplastic materials like tri-calcium phosphate (TCP) are viable substitutes for autogenous bones or other bone substitutes [9]. When blended with bone substitutes, the ability of PRF for regeneration is enhanced. The Recombinant human bone morphogenetic protein-2 (rhBMP-2) is promising in various bone augmentation procedures [10]. An animal study has reported unique bone formation escorted with rhBMP-2 impregnated β-TCP scaffold in the cranial bone defect model [11]. The utility of rhBMP-2 and Acellular Collagen Sponge (ACS) in extraction defects helps to reconstruct the buccal plate. The alveolar ridge dimensions are crucial for implant placement, and rhBMP-2 aids in maintaining ridge configuration favoring implant placement [12]. Similarly, the crucial bone structure inherent in the healing socket is also important. Clinically, radiographically and histologically, the efficacy of ridge preservation using rh-BMP-2 in conjunction with β-TCP and PRF was therefore evaluated.

## Main text

### Materials and method

Following informed consent, two systemically healthy patients (M:F1:1, aged 23 years and 30 years, respectively) who required the extraction of teeth for reasons other than periodontitis were recruited (Additional file 1: Fig S1, Additional file 2: Fig S2). Initial treatment, consisting of oral hygiene instructions, supragingival and subgingival scaling, was done after careful assessment and diagnosis. The full mouth plaque index (FMPI, [13]) and Full mouth papillary bleeding index (FMPBI) scores were low at the baseline and remained low (< 1) during the treatment period (Table 1) [14]. We included two patients with four sites. This was accompanied by a clinical and radiographic analysis of the chosen areas (Table 2).

**Table 1:** Full Mouth Papillary Bleeding Index (FMPBI) and Full Mouth Plaque Index (FMPI)

Cases	FMPBI at Baseline	FMPBI at 3 months	FMPI at Baseline	FMPI at 3 months
Cases 1	0.5	0.4	0.4	0.4
Cases 1	0.5	0.3	0.4	0.3
Cases 2	0.5	0.5	0.5	0.5
Cases 2	0.5	0.5	0.5	0.5
Mean ± SD	0.5 ± 0	0.45 ± 0.05	0.425 ± 0.08	0.425 ± 0.08
p-value	0.215 NS		0.391 NS	

*NS* not significant

**Table 2:** Measurements at ridge prior tooth/root piece extraction (at baseline) and at 3 months (Post-operative)

		At baseline					At 3 months			Mean ± SD (Bone loss)			
Cases	Site	Mesio-distal width (mm)	Bucco lingual width (mm)	Measurement of root piece (mm)		Distance from root apex to anatomical structures (mm)	Mesiodistal width (mm)	Buccolingual width (mm)	Apico coronal length (RBH,mm)	M-D (mm)		B-L (mm)	
										At Baseline	At 3 months	At Baseline	At 3 months
										9.6 ± 1.67	9.0 ± 1.54	8.4 ± 1.13	7.9 ± 1
Cases 1	11	8.5	7.5	12		3	8	6.5	14.5	Mean Difference (Bone Loss,mm) = 0.6 ± 0.13		Mean Difference (Bone Loss,mm) = 0.5 ± 0.13	
Cases 1	12	7.5	7	11		3	7	7	13.5				
Cases 2	46	11.5	9.5	MB-8	DB-6	4	10.5	9	12	p = 0.015*		p = 0.058 NS	
Cases 2	47	11	9.5	MB-7	DB-7	5	10.5	9	12.5				

*NS* not significant

* Statistically significant

#### Surgical procedure

A pre-surgical rinse with chlorhexidine gluconate 0.12% mouthwash (Rexidine®, Indoco Remedies Ltd) for 2 min was advocated. After administering local anesthesia (Xicaine®, ICPA Health Products Limited), flapless minimally invasive periotomy was carried out with a Bard-Parker Surgical blade number #15 (Glassvan®, Niraj Industries Pvt Ltd) to facilitate atraumatic extraction without causing damage to the buccal plate (Additional file 3: Fig S3). The walls of the socket were consecutively tested for integrity. Twenty milliliters of venous blood is removed from the antecubital vein in two test tubes. Centrifugation of the blood was carried out at 3000 rpm for 12 min to obtain PRF. [15] One part of PRF was mixed with β-TCP (OSTEON TM II Genoss Co., Ltd, Korea) and 1.5 mg/mL concentration of rhBMP-2 (GibcoR, Recombinant Human Bone Morphogenetic protein-2, Life Technologies, Van Allen Way, Carlsbad, California,16) to enhance wound healing. The socket was filled up to the level of the alveolar crest. The remaining clot was compressed to a membrane of high tensile strength. The membrane was then carefully placed, covering the graft material. The approximation of the surgical site was made using a 3–0 silk suture. (Centisilk Non-Absorbable Surgical Suture USP) (Additional file 4: Fig S4).

**Fig. 1:**
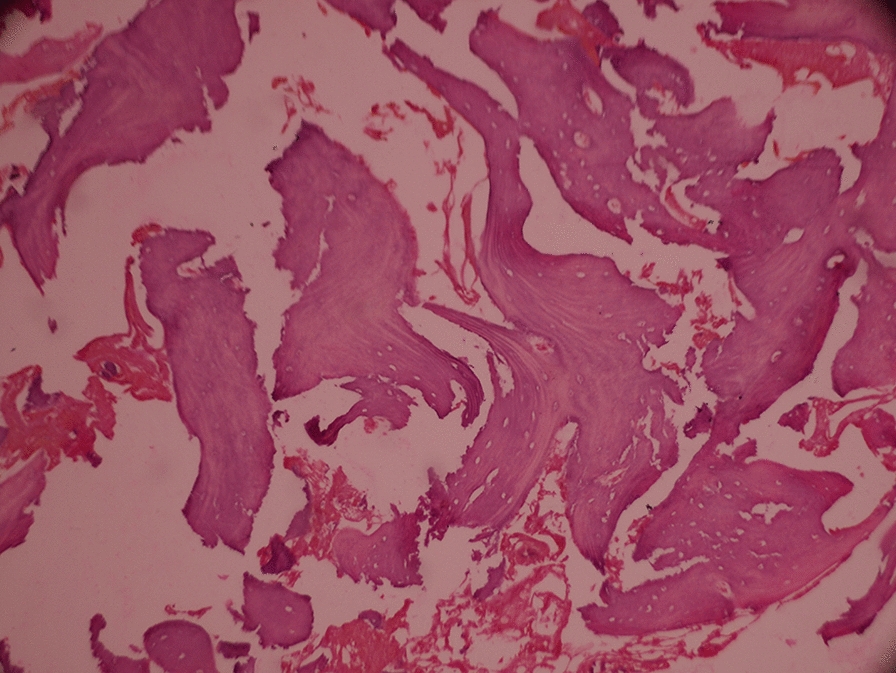
Histological view of new bone formation 3 months after ridge preservation

The patients were checked for implant placement after three months (Additional file 5: Fig S5). Following administration of local anesthesia (Xicaine®, ICPA Health Products Limited), an incision was given from the line angle of adjacent teeth to elevate the mucoperiosteal flap and expose the underlying bone (Additional file 6: Fig S6). Alveolar ridge measurements were taken intrasurgically and were compared with the pre-operative alveolar dimensions (Table 2). A 2 mm trephine drill was initially used to harvest bone core for histological examination. The bone tissue was moved to a mixture containing 10% neutral buffered formalin. A decalcification protocol was used to remove the bone mineral component to ease the fine section and better study of new bone-forming cells. The tissues were stained with Hematoxylin & Eosin (H & E, Fig. 1, [17]).

Sequential drills were used for osteotomy preparation, based on the size of the implant selected. An implant (Equinox, Myriad Plus TM implant system) of the largest possible dimension was placed according to the manufacturer's recommendations, and further follow-up was carried out. Four implants were placed in two patients (Additional file 7: Fig S7, Additional file 8: Fig S8).

#### Statistical analysis

The Wilcoxon sign ranked test was used to compare the means of the parameters used before and after socket grafting. A p < 0.05 was considered to be statistically significant. [18].

### Discussion

For peri-implant soft tissues, the healthy osseous contour creates a skeleton. Atraumatic extraction helps to eliminate bone resorption and preserve the dimensions of the alveolar ridge during healing. In the present study, three months following socket preservation, minimal changes in the alveolar ridge dimensions were seen (mesiodistal bone loss 0.6 ± 0.13 mm, and buccolingual bone loss 0.5 ± 1.13 mm), and the results were not significant. The physiological hardness of the bone measured before implant insertion during drilling produced a tactile feeling similar to drilling into spruce or white pine wood in all four positions [19]. Although bone density evaluation using Hounsfield units was not done, dense to the thick porous cortical bone on the crest and coarse trabecular bone underneath was seen on radiographic examination, suggesting D2 bone quality [20]. Histologically, the presence of essential mineralized osteoid trabeculae and spindle nuclear cell soft tissue revealed new bone structure. All four implants achieved good primary stability. As soft tissue grows six times faster than the bone tissue [21], in the present study, a saddle of PRF membrane covering the socket helps prevent the gingival connective tissue down-growth. It also held the osseous graft in place [22]. Additionally, the membrane also aided in clot stabilization. PRF principally contains a fibrin matrix rich in platelets and leukocytes. Cytokines such as IL-1β, -4, and -6, and growth factors such as PDGF, TGF-beta, IGF, EGF, and VEGF are other vital components present. These are the critical elements in bone regeneration [23]. Fibrin gel in coagulation cascade aids in fibrinogen molecules collaboration and formation of a highly biocompatible three-dimensional fiber network [24]. PRF has also contributed to accelerated wound healing and increased bone graft density and maturation [19]. β-TCP is (purified, microcrystalline porous) a form of calcium phosphate. Remarkable resorption of β-TCP particulate is estimated at around 3 to 6 months after placement. In addition, particles are well absorbed into the current formation of bones, forming a thick cancellous matrix. Biodegradation of graft takes place both by osteoclastic activity as well as chemical dissolution by tissue fluids. A solid new bone formation was evident during implant placement, which favored the placement of ideal implant size. The use of rhBMP-2 necessitates the use of a carrier for its osteoinductivity [25]. Misch emphasized the advantages of rhBMP-2 in new bone growth in patients requiring extraction. In the present study, rhBMP-2 was used as an agent to promote socket repair and aid in ridge augmentation when combined with a part of β -TCP. Fiorellini et al. [16] in their study showed significantly greater bone augmentation with 1.50 mg/mL/rhBMP-2 compared to a concentration of 0.75 mg/mL in socket defects. Therefore, in the present study, a concentration of 1.50 mg/mL/rhBMP-2 was used. Various studies [26, 27] have supported using a mixture of β-TCP and rh-BMP-2 to facilitate the increased bone formation; hence rh-BMP-2 was used in combination with β-TCP to promote bone growth.

## Limitations

The present analysis explains in a small sample the findings obtained. Although there is evidence of good-quality bone formation during a short period following the use of a combination of rhBMP-2, β-TCP, and PRF, more extensive population studies may further improve the significance of the results obtained. PRF was used to cover and hold the graft in place. The increase in keratinized tissue thickness following the PRF membrane placement was not evaluated. In addition, follow-up outcomes have been recorded for just six months (Additional file 9: Fig S9); additional long-term patient monitoring will be helpful. Even though pre-operative CBCT was taken but due to the patient's cost concerns and unwillingness, the post-operative CBCT could not be accepted.

## Supplementary Information

**Additional file 1: Fig S1.** Pre-Operative Clinical view.

**Additional file 2: Fig S2.** Pre-operative Radiographic view.

**Additional file 3: Fig S3.** Extracted root piece and tooth.

**Additional file 4: Fig S4.** Approximation after Ridge/socket preservation.

**Additional file 5: Fig S5.** Clinical view of edentulous site 3 months after ridge preservation.

**Additional file 6: Fig S6.** Clinical view after mucoperiosteal flap reflection.

**Additional file 7: Fig S7.** Implants in position.

**Additional file 8: Fig S8.** After final prosthesis placement.

**Additional file 9: Fig S9.** Radiograph showing implant in position at six months follow up.

## Data Availability

The datasets used and/or analysed during the current study are available from the corresponding author on reasonable request.

## Abbreviations

rhBMP: 2-recombinant bone morphogenetic protein
β-TCP: Beta-tricalcium phosphate
PRF: Platelet rich fibrin
PDGF: Platelet-derived growth factor
TGF: Beta transforming growth factors
IGF: The insulin-like growth factor
EGF: Epithelial growth factor
VEGF: Vascular endothelial growth factor
FMPBI: Full Mouth Papillary Bleeding Index
FMPI: Full Mouth Plaque Index
MB: Mesio-buccal root
DB: Disto-buccal root
RBH: Residual bone height-measured from alveolar crest to anatomical structure
M-D: Mesiodistal width
B-L: Buccolingual width
