# Supplementary figures and images for: Extraction socket grafting using recombinant human bone morphogenetic protein-2-clinical implications and histological observations
Source: BMC Res Notes. 2021 Feb 15;14:61. doi: 10.1186/s13104-021-05476-0 (PMC7903772; doi:10.1186/s13104-021-05476-0)

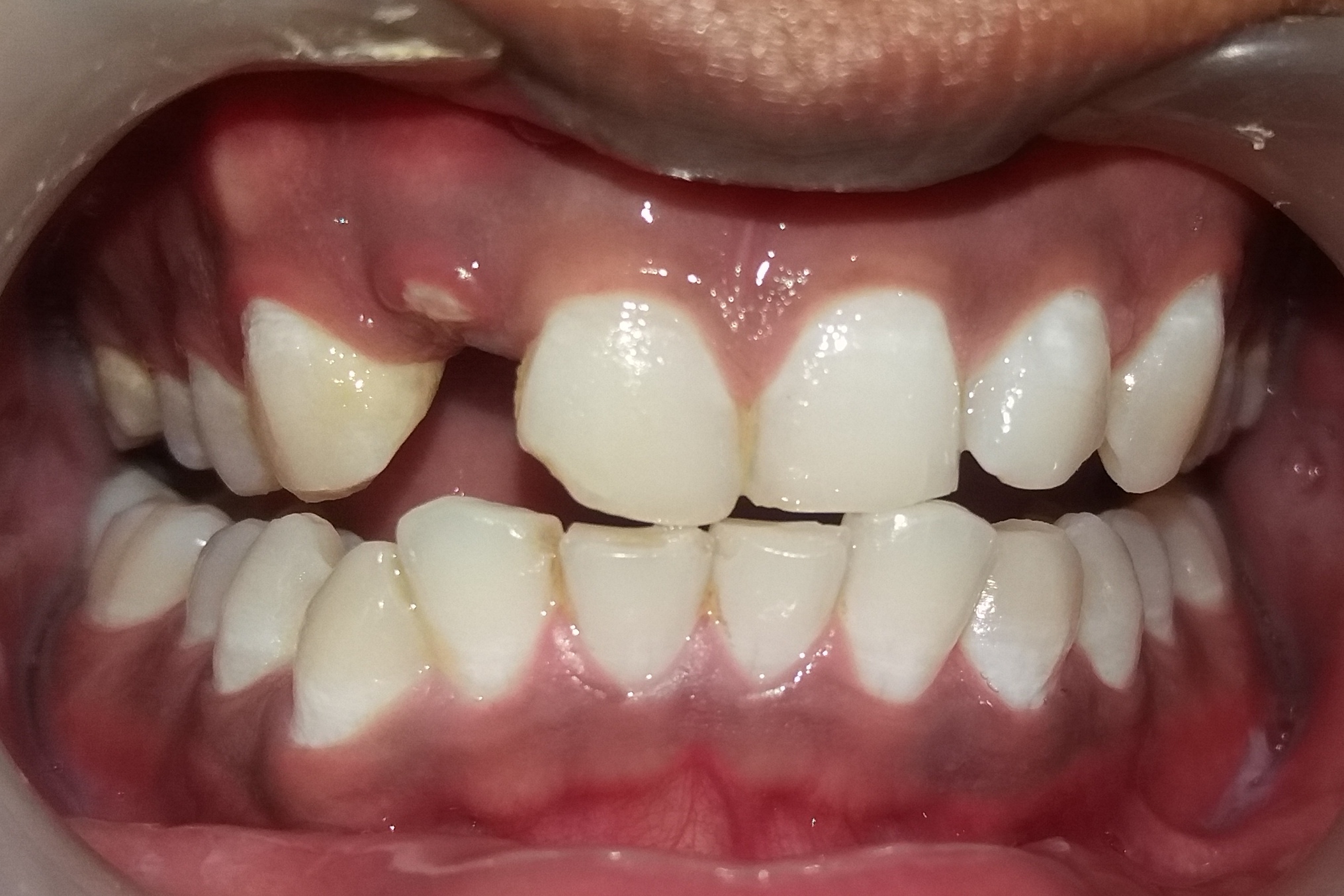

Supplement: Supplementary file 1 — Additional file 1: Fig S1. Pre-Operative Clinical view. [file 13104_2021_5476_MOESM1_ESM.jpg]

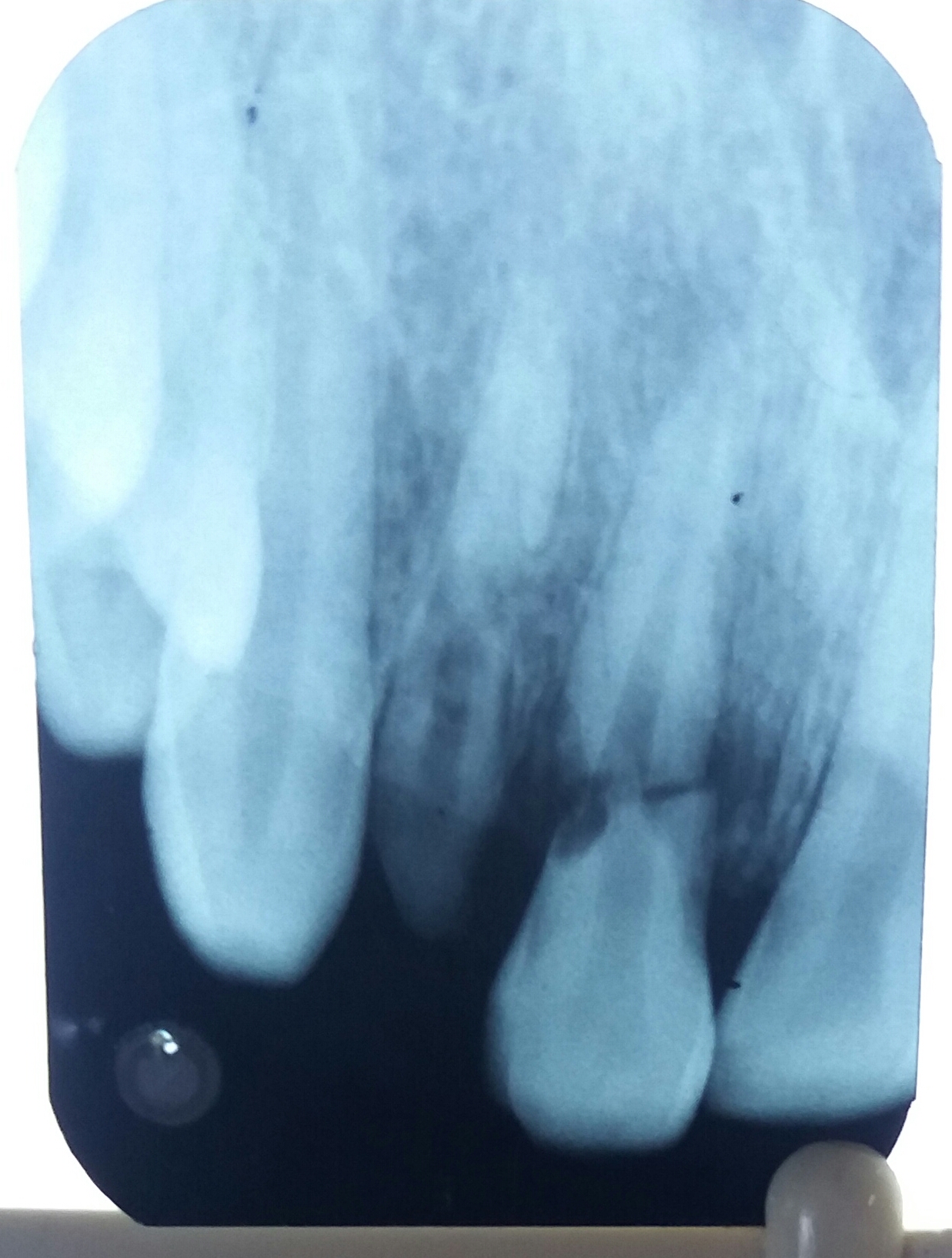

Supplement: Supplementary file 2 — Additional file 2: Fig S2. Pre-operative Radiographic view. [file 13104_2021_5476_MOESM2_ESM.jpg]

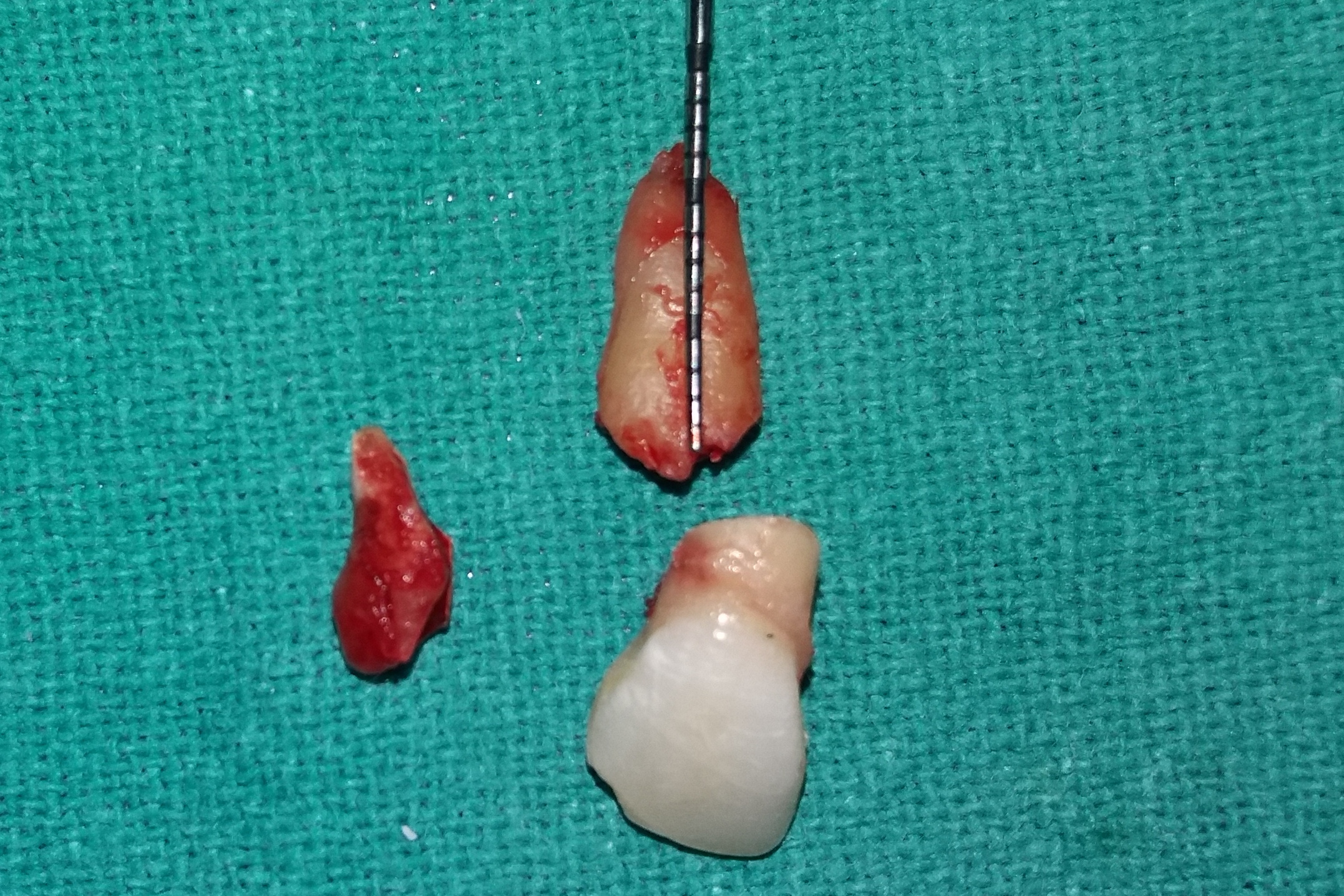

Supplement: Supplementary file 3 — Additional file 3: Fig S3. Extracted root piece and tooth. [file 13104_2021_5476_MOESM3_ESM.jpg]

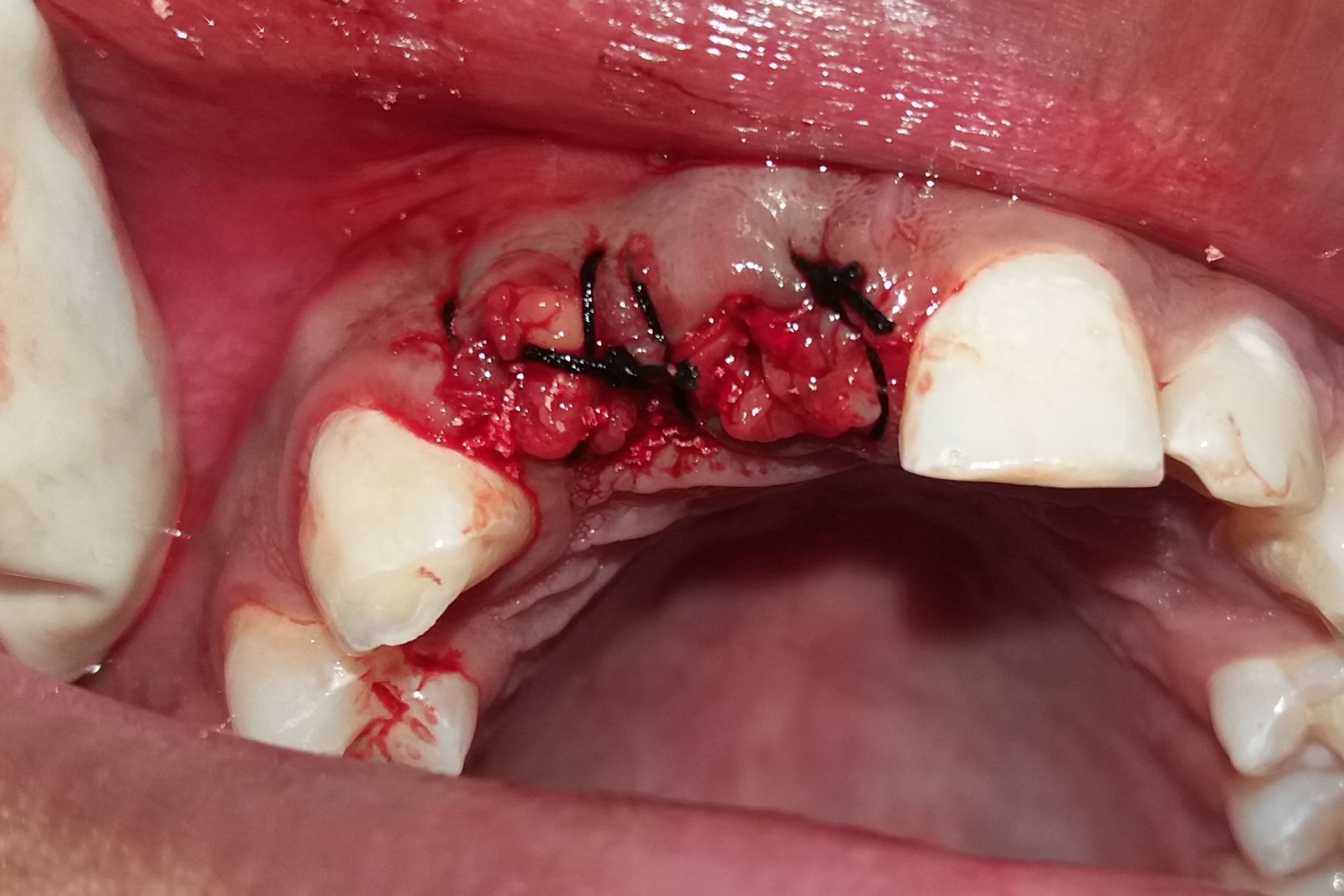

Supplement: Supplementary file 4 — Additional file 4: Fig S4. Approximation after Ridge/socket preservation. [file 13104_2021_5476_MOESM4_ESM.jpg]

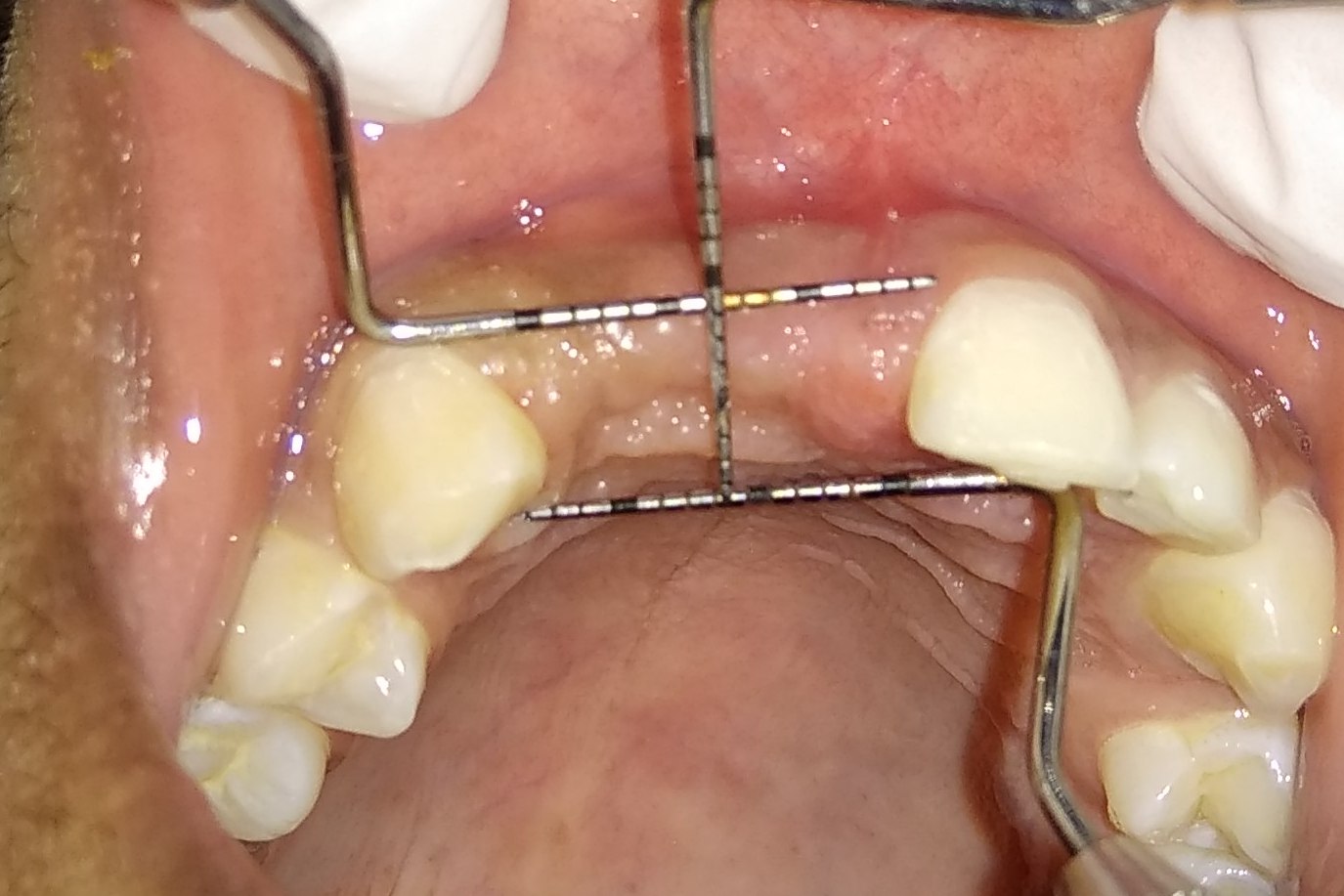

Supplement: Supplementary file 5 — Additional file 5: Fig S5. Clinical view of edentulous site 3 months after ridge preservation. [file 13104_2021_5476_MOESM5_ESM.jpg]

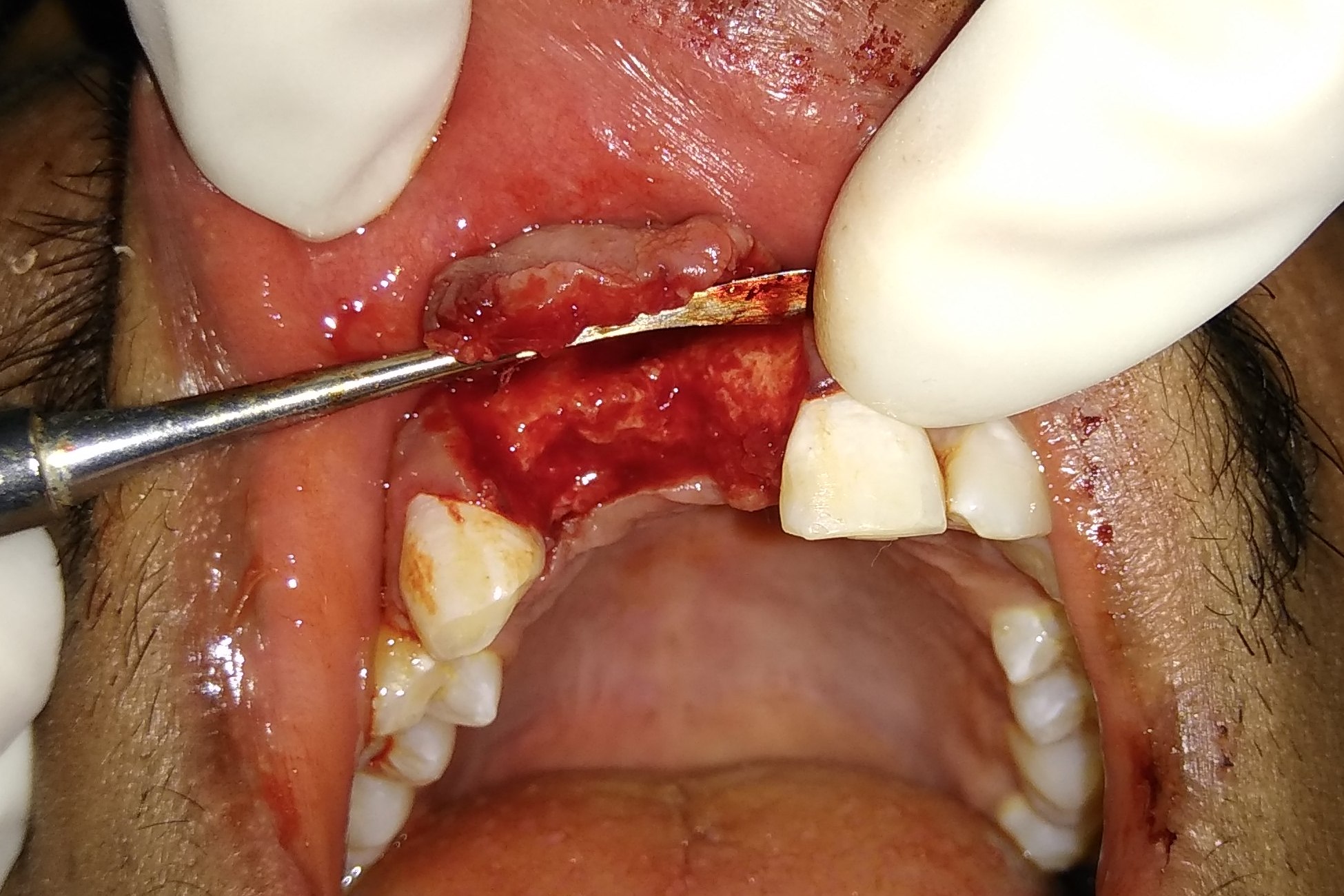

Supplement: Supplementary file 6 — Additional file 6: Fig S6. Clinical view after mucoperiosteal flap reflection. [file 13104_2021_5476_MOESM6_ESM.jpg]

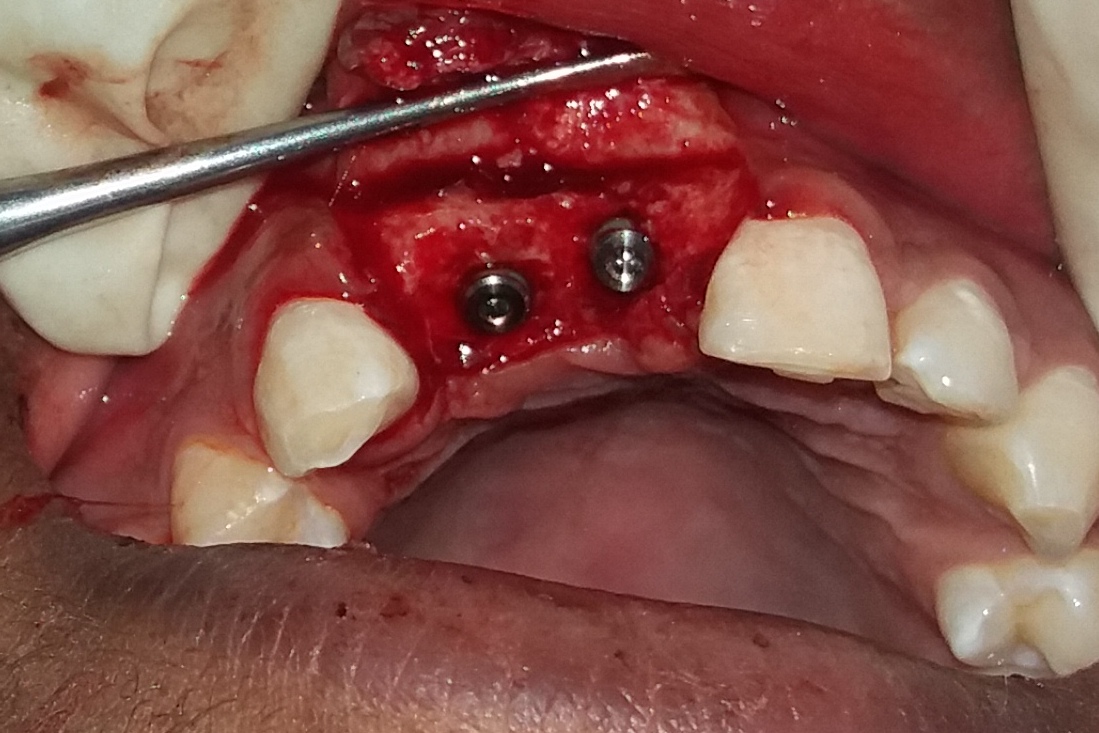

Supplement: Supplementary file 7 — Additional file 7: Fig S7. Implants in position. [file 13104_2021_5476_MOESM7_ESM.jpg]

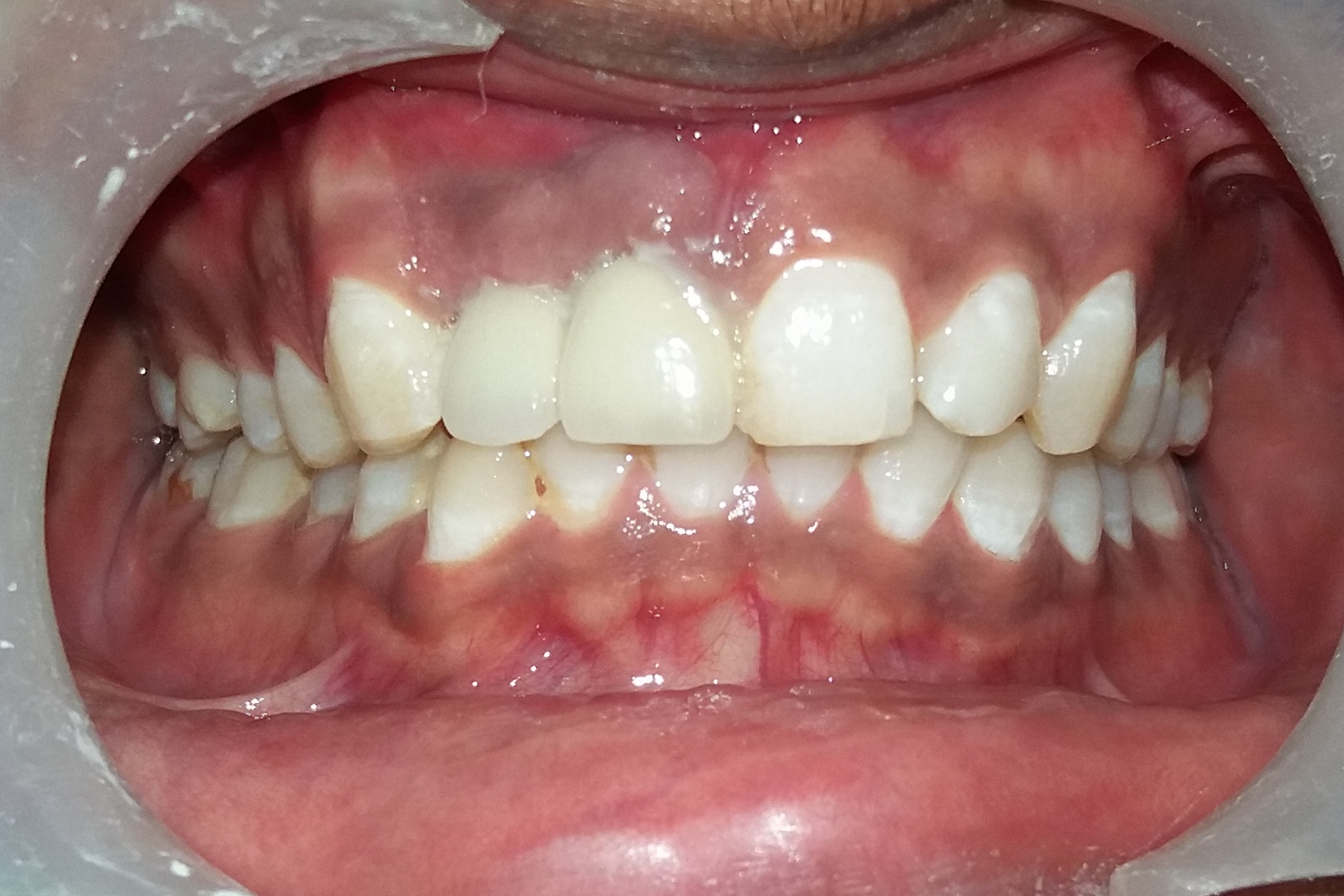

Supplement: Supplementary file 8 — Additional file 8: Fig S8. After final prosthesis placement. [file 13104_2021_5476_MOESM8_ESM.jpg]

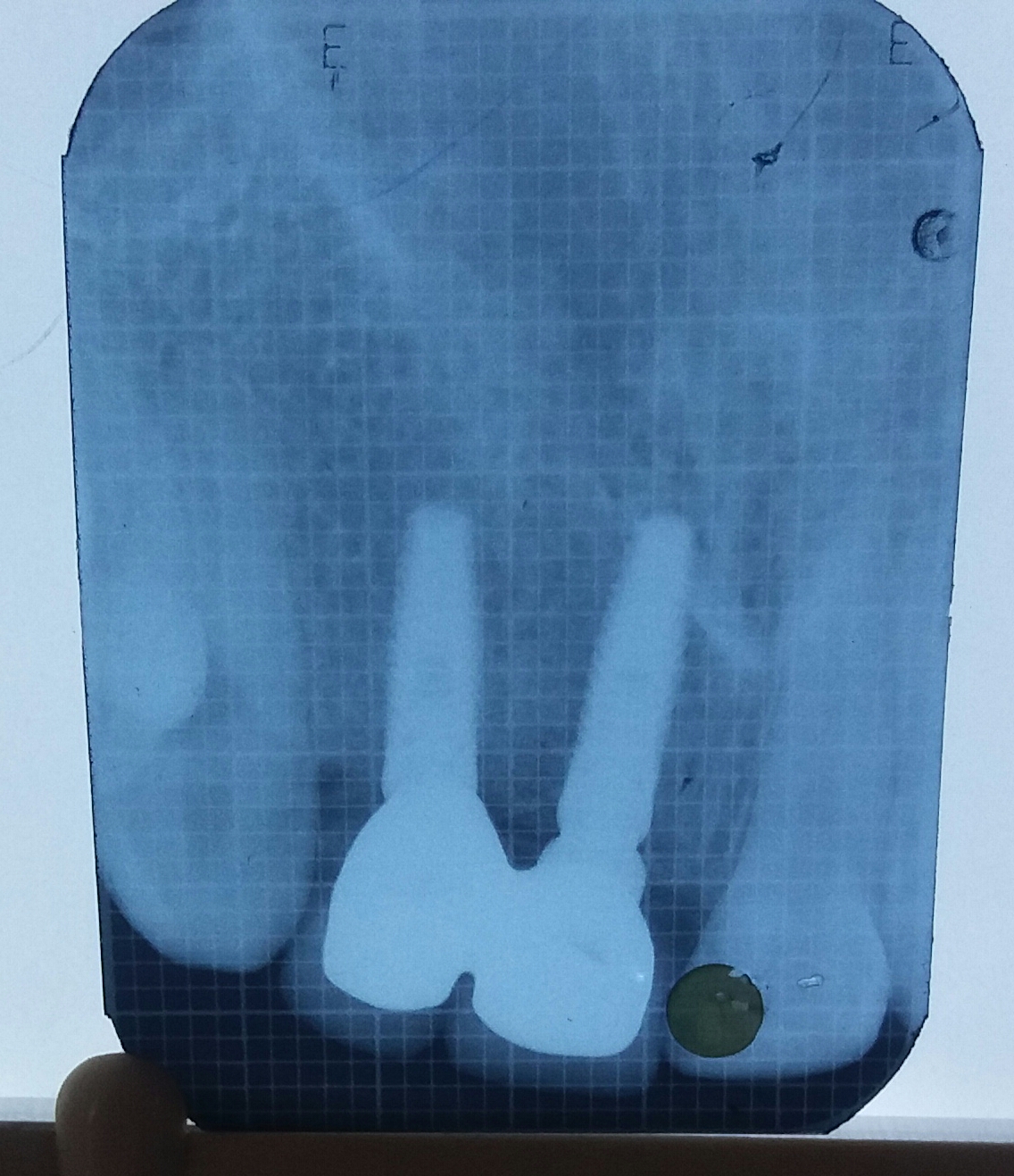

Supplement: Supplementary file 9 — Additional file 9: Fig S9. Radiograph showing implant in position at six months follow up. [file 13104_2021_5476_MOESM9_ESM.jpg]
